# Free versus Pedicled Flaps for Lower Limb Reconstruction: A Meta-Analysis of Comparative Studies

**DOI:** 10.3390/jcm11133672

**Published:** 2022-06-25

**Authors:** Matteo Scampa, Vladimir Mégevand, Domizio Suva, Daniel F. Kalbermatten, Carlo M. Oranges

**Affiliations:** 1 Department of Plastic, Reconstructive and Aesthetic Surgery, Geneva University Hospitals, Geneva University, 1205 Geneva, Switzerland; vladimir.megevand@gmail.com (V.M.); daniel.kalbermatten@hcuge.ch (D.F.K.); 2 Department of Plastic Surgery, Guy’s and St Thomas’ NHS Foundation Trust, St Thomas’ Hospital, London SE1 7EH, UK; 3 Bone Infection Unit, Department of Orthopedic Surgery, Geneva University Hospitals, University of Geneva, 1205 Geneva, Switzerland; domizio.suva@hcuge.ch

**Keywords:** free flap, pedicled flap, meta-analysis, outcomes, comparative, lower limb

## Abstract

Background: Free and pedicled flaps are both valuable surgical strategies for lower limb reconstruction. Evidence that compares both techniques is scarce. Our aim is to synthetise all the comparative studies by conducting a meta-analysis to identify post-operative outcomes. Method: A systematic review of pubmed, EMBASE, Cochrane library, and Web of Science was conducted, aiming at articles comparing the outcomes of free versus pedicled flaps in lower limb reconstruction. A pooled analysis with the Mantel and Haenszel methods and random effect analysis provided results as a risk ratio with a 95% confidence interval. Results: 10 retrospective studies were selected. While the flap necrosis rate did not differ significantly between techniques (RR 1.35, 95%CI 0.76–2.39, *p* = 0.31), the partial flap necrosis rate was significantly lower in free flaps (RR 0.45, 95%CI 0.22–0.91, *p* = 0.03). The overall complication rate (RR 0.83, 95%CI 0.64–1.07, *p* = 0.16) and revision surgery rate (RR 1.38, 95%CI 0.55–3.50, *p* = 0.49) did not differ significantly. No significant difference was found in the high aesthetic satisfaction rate (RR 1.76, 95%CI 0.57–5.41, *p* = 0.32) and post-operative infection rate (RR 0.85, 95%CI 0.55–1.33, *p* = 0.48). Conclusion: Despite important variability in the choice of flaps and outcomes reported among studies, free and pedicled flaps appear to be reliable surgical strategies for lower limb reconstruction with similar surgical outcomes.

## 1. Introduction

Lower limb reconstruction is a complex task requiring a multidisciplinary approach. It requires plastic surgery and orthopaedic skills to cope with soft tissue defects, bone loss, and fractures from diverse aetiologies ranging from trauma to chronic wounds. The orthoplastic concept has been implemented in reference centres to improve the success of the procedures, as failure can have dramatic functional and aesthetic outcomes such as limb amputation [1,2,3]. Difficulties come from the paucity of soft tissues, proximity between the skin, and the underlying profound structures such as bone and tendons limiting the possibility of primary closure and wound healing by secondary intention [4]. Flaps allow surgeons to cope with these difficulties by transposing well vascularized soft tissues from a healthy area to the wound. Lower limb flap reconstruction is considered to be technically more challenging due to frequent vascular injury, potential venous congestion, and the distribution of weight bearing zones [5,6,7]. The pedicled flap (PF) choice remains limited in this area and free flaps (FF) exist as an important adjunction to the plastic surgeon’s armamentarium due to the multitude of donor-sites available. FF are considered the gold standard for large wounds associated with extended damage in local tissues and are often the only available option [8]. However, these microsurgical procedures are complex, time and resource consuming, and require expertise to avoid flap loss due to vascular compromise.

The aim of this meta-analysis is to synthetise all evidence comparing FF and PF in the lower limb to define the advantages/disadvantages of each technique. Our aim is to help guide plastic surgeons in choosing the most adapted surgical strategy for their patients depending on their needs and expectations.

## 2. Materials and Methods

This meta-analysis follows the PRISMA 2020 guidelines for reporting meta-analyses [9].

*Search strategy:* A systematic review of Pubmed, EMBASE, Cochrane library, and Web of Science was conducted on the 1 March 2022 seeking for all studies comparing outcomes between FF and PF in lower limb reconstruction. Search strategy was defined by following the PICO principles and detailed in Table 1. 

*Article selection:* Inclusion and exclusion criteria were defined using PICO before conducting the systematic review. We included all comparative studies in the English language comparing lower limb reconstructions between FF and PF. Exclusion criteria were unpublished studies, animal studies, and studies not reporting the main outcome (Table 2).

Articles retrieved from the search strategy were independently screened by title and abstract by two authors (M.S.; V.M) using the Rayyan software for systematic review (https://www.rayyan.ai/; accessed on 1 March 2022) [10]. In the case of a discordant decision, article was screened by a third author (C.M.O) and decision was taken after concertation between the 3 authors. A secondary manual search was conducted on Research Gate and Google Scholar to retrieve articles potentially missed during the systematic review.

Selected articles were then fully read. If they met all the inclusion criteria, data was extracted independently by two authors (M.S.; V.M) and processed using Review Manager (RevMan) (V5.4.1, The Cochrane Collaboration, 2020). No attempt to retrieve missing data was performed.

*Outcomes:* Primary outcome was flap necrosis rate and had to be reported in all selected articles. Secondary outcomes were partial flap necrosis rate, overall complication rate, revision surgery rate, post-operative wound infection rate, and high aesthetic satisfaction rate. If definition of outcome between selected articles was not similar, data was included in statistical analysis, but a footnote had to specify the definition. The overall complication rate includes all reported complications of donor and recipient site, including flap loss. Outcomes had to be reported in more than 3 studies to be included in the meta-analysis.

*Statistical analysis:* Differences in outcomes between intervention were expressed as Risk Ratio (RR), with a 95% confidence interval (CI). The Mantel and Haenszel methods were used to combine studies’ results with a random effect analysis, because variability inside and between studies was expected to be high. Heterogeneity was expressed using the I2 statistic, with values below 30% considered as low heterogeneity and over 70% as high heterogeneity [11]. A leave-one-out test, consisting of calculating the pooled risk ratio by sequentially excluding one study, was performed to identify studies with a strong influence on the results.

## 3. Results

**Figure 1:**
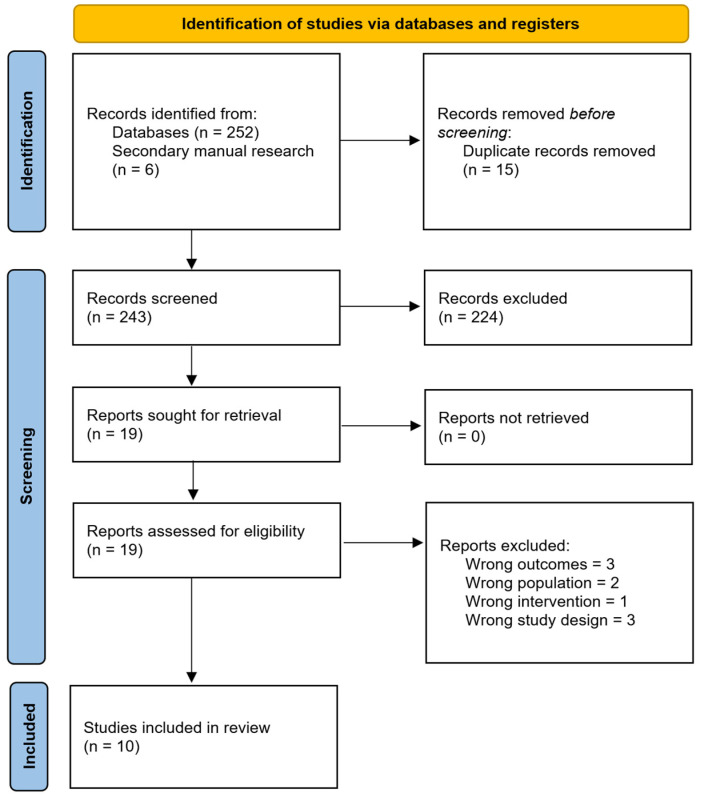
Systematic review flow chart.

Two-hundred-fifty-two studies were retrieved with the systematic review and six were retrieved during the secondary manual research. Fifteen duplicates were eliminated. After being screened by title and abstract, only 19 studies were considered eligible for complete reading. Ten studies met all the selection criteria [12,13,14,15,16,17,18,19,20,21] (Figure 1).

The 10 studies were of a retrospective nature and aimed at comparing different outcomes between FF and PF of the lower limb. All the studies were mono-centric except Koh et al., which was multi-centric and international (Korea, Singapore, USA) [17]. The meta-analysis covers a total of 1229 procedures, of which were 795 FF and 434 PF (Table 3). Most of the studies are recent, except for Ducic et al. and Zook et al., who cover a study period before the year 2000 [14,20]. The type of flaps between each study varied greatly, including fascio-cutaneous, musculo-cutaneous, and muscular FF and PF ranging from local perforator propeller flaps to pedicled musculo-cutaneous flaps. However, the outcomes in these studies were always compared between FF and PF. While some studies limited the analysis to only one flap per patient, some included multiple flap procedures per patient. Population age was heterogeneous between studies, with four studies including pediatric patients [16,18,19,20]. Defect aetiology varied greatly between and inside the selected studies including traumatism, neoplasm, infections, scar contractures, and chronic wounds (with peripheral vascular disease, irradiation, and neuropathy).

**Figure 2:**
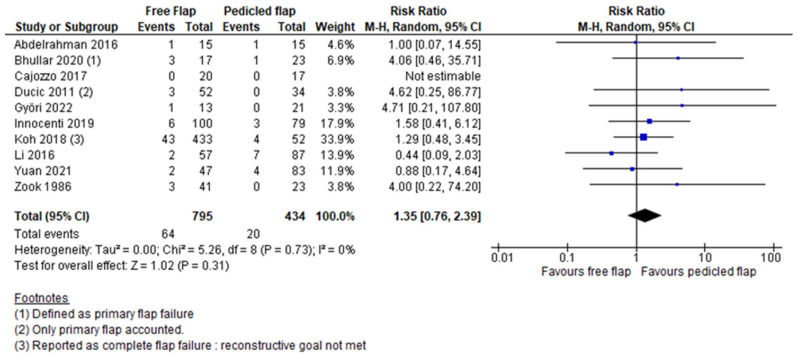
Total flap necrosis meta-analysis [12,13,14,15,16,17,18,19,20,21].

**Figure 3:**
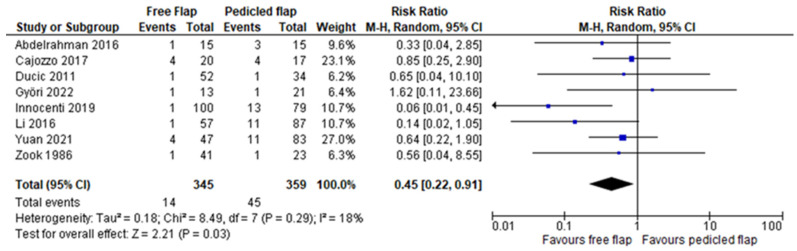
Partial flap necrosis meta-analysis [12,13,14,15,16,18,19,20].

**Figure 4:**
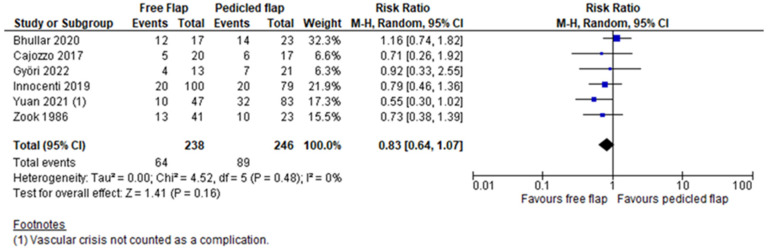
Overall complications meta-analysis [13,15,16,19,20,21].

**Figure 5:**
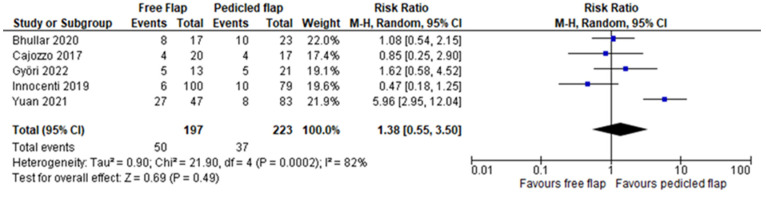
Revision surgery meta-analysis [13,15,16,19,21].

**Figure 6:**
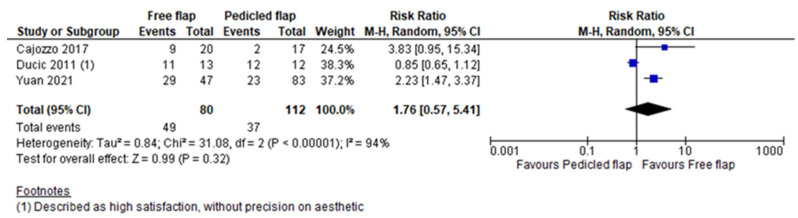
Patient high aesthetic satisfaction meta-analysis [13,14,19].

**Figure 7:**
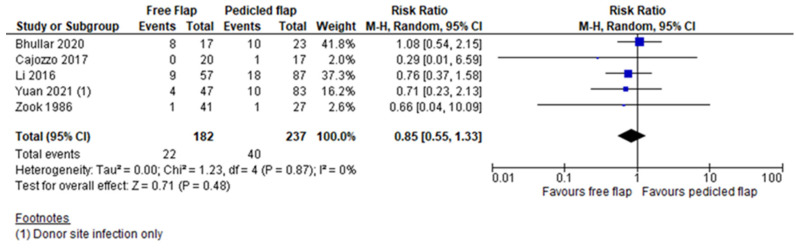
Post-operative wound infection meta-analysis [13,18,19,20,21].

Complete flap necrosis was reported in all 10 articles and the pooled analysis did not find a significant difference between FF and PF (RR 1.35, 95%CI 0.76–2.39, *p* = 0.31) [12,13,14,15,16,17,18,19,20,21]. (Figure 2) A partial flap failure was reported in eight articles and FF had a significantly lower risk than PF in the pooled analysis (RR 0.45, 95%CI 0.22–0.91, *p* = 0.03) [12,13,14,15,16,18,19,20] (Figure 3). Overall complication rates were reported in six studies, and the pooled analysis found no significant difference between FF and PF concerning the overall risk of complications (RR 0.83, 95%CI 0.64–1.07, *p* = 0.16) [13,15,16,19,20,21] (Figure 4). For Yuan et al.’s work, a vascular crisis was not counted as a complication, as it was defined as a clinical sign motivating surgical exploration. Only adverse outcomes from this surgical exploration were accounted in the overall complication rate [19]. Revision surgery rates were reported in five studies, and no significant difference in risk was found between FF and PF (RR 1.38, 95%CI 0.55–3.50, *p* = 0.49) [13,15,16,19,21] (Figure 5). Revision surgeries were reported for functional purposes such as skin grafting, debridement, etc. The patient aesthetic satisfaction was assessed in 3 studies, and no significant difference in high satisfaction rate was found between FF and PF (RR 1.76, 95%CI 0.57–5.41, *p* = 0.32) [13,14,19]. (Figure 6). The post-operative wound infection rate was reported in five studies, without finding a significant difference in the pooled analysis between FF and PF (RR 0.85, 95%CI 0.55–1.33, *p* = 0.48) [13,18,19,20,21] (Figure 7). The heterogeneity between studies was low (I2 < 30%), except for the revision surgery and aesthetic satisfaction rates where it was considered high (I2 > 70%).

By assessing the leave-one-out analysis, we noted that Innocenti et al. and Li et al.’s studies strongly favoured FF with respect to the partial flap necrosis rate and their exclusion resulted in non-significant results [16,18] (Table 4). Bhullar et al. strongly influenced the overall complication rate by counterbalancing other studies that favoured FF, with its exclusion leading to statistically significant results [21]. Ducic et al. also had a strong influence in aesthetic outcome by favouring PF [14].

## 4. Discussion

This study is to our knowledge the first meta-analysis of comparative studies between FF and PF. It shows that FF is a reliable surgical strategy for coping with lower limb soft tissue defect with complete necrosis rates similar to PF. Our initial hypothesis was that FF might have a higher complete necrosis rate due to the microsurgical anastomosis compared to PF where no anastomosis is required. While FF’s necrosis rate was higher, the difference was not statistically significant. Interestingly, Li et al. reported a higher prevalence of complete necrosis in PF [18]. They explain this phenomenon by a strong association between post-operative infection and PF necrosis, but also by flap-related factors such as flap size [18]. However, even by excluding this article from the meta-analysis the results remain non-significant.

However, partial flap necrosis was significantly higher in the PF group. This result can be explained by the inclusion of propeller flap. They are PFs with a skin paddle that relies on a perforator which acts as point of rotation. If the flap size is too large, the flap extremity can be exposed to partial necrosis. Innocenti et al. found high partial flap necrosis rates in a population including only propeller flaps; however, complete necrosis remained rare [16]. This observation can be explained by the use of a reliable perforator but experiencing difficulties with defining the maximal flap size that can be harvested on a single perforator. Cajozzo et al. also compared propeller flaps to FFs and found an even higher partial necrosis rate (23%) but a similar rate in the FF group [13]. The higher FF partial necrosis rate in this study might be explained by the inclusion of all kinds of FF, such as muscular and fasciocutaneous ones, compared to only perforator fascio-cutaneous FF in Innocenti’s work [13,16]. Other studies included different PFs and found fewer radical results. The impact of Innocenti et al.’s study is important as its exclusion from the meta-analysis resulted in non-significant results [16]. The use of intra-operative indocyanine green (ICG) might help detect the compromised vascularization of flaps [22]. However, none of the studies included in the meta-analysis described the use of ICG.

The overall complication rate was found to be similar between FF and PF. All the studies showed similar results [13,15,16,19,20,21]. Unfortunately, donor site complications were not reported or were reported inconstantly in the selected studies, thereby not allowing for the inclusion of this variable in this meta-analysis. On the other hand, the results regarding revision surgery rates were more mitigated between studies. Innocenti et al. reported higher rates of revision surgery in pedicled propeller flaps, while Yuan et al. found more revisions in the FF group [16,19]. Yuan et al. explain it via the presence of a high rate of vascular crises (7 out 47) requiring revision and the inclusion of secondary procedures such as flap thinning due to excessive fatty tissue in thigh FF [19]. However, secondary procedures can help optimize the functional and aesthetic outcomes [23]. The sequential exclusion of those studies does not result in a statistically significant difference of the revision rates between FF and PF. These results should be interpreted cautiously due to a high bias potential because the definition of revision surgery is not similar between the studies and the indications were not sufficiently described.

Patient satisfaction rate seems to favour FF, despite no statistical difference. While Yuan et al. and Cajozzo et al. specifically analysed aesthetic satisfaction, Ducic et al. included the overall patient’s satisfaction and found results to be more mitigated [13,14,19]. The exclusion of this study allows one to find significant results in favour of FF [14]. FF offers the choice of multiple donor sites that allows for the acquisition of matching skin and pilosity patterns. Furthermore, the use of fascio-cutaneous flaps provides thin and smooth skin paddles identical to the lower limb cutaneous aspect [24]. Kotsougiani et al. found a high overall satisfaction for FF procedures with a modest cosmetic satisfaction, inversely corelated with the number of secondary procedures in a retrospective cohort of 389 patients [25]. Interestingly, the necessity of secondary refinement surgery was low (13.9%) [25].

The post-operative wound infection rate did not differ significantly between FF and PF in the pooled analysis. However, post-operative infection rates varied consistently between studies. Bhullar et al. also reported high infection rates (47% FF/43% PF) [21]. Those rates can be explained by the study population, which comprises only complex open fractures rated Gustillo 3 [21]. Open fractures are often high-energy injuries and are associated with vascular damage and wound contamination, with the proportion of infections rising with the Gustillo classification [26]. Li et al. reported high post-operative infection rates (16% FF/21% PF) identified by positive bacteriological analysis [18]. They explain these results by association with pre-operative wound bed inflammation and the timing of the reconstruction with sub-acute reconstruction (>72 h, <90 days) being associated with more infections [18]. Interstudy variability can be explained by the different definitions of infection and different populations, suggesting that the pooled results might be unreliable. Furthermore, some studies reported distinct donor site infection, while other reported an overall infection rate.

This meta-analysis exposes interesting results; however, they are results that need to be interpreted with care due to potential bias. One limitation is the diversity of the studies’ populations with defects from different aetiologies. The type of wound can impact surgical outcomes such as in the post-operative infection rate. Furthermore, no distinction between the types of tissues included in the flaps was assessed. While the studies in this meta-analysis included muscle and fascio-cutaneous flaps in each study arm, Bekkara et al.’s meta-analysis included only fascio-cutaneous pedicled flaps in the comparative arm (pedicled flaps) and found similar results with higher partial flap necrosis in the pedicled flap arm and no significant difference in the complete flap failure or overall complication rates [27]. Differences between muscular and fascio-cutaneous free flaps have already been investigated by Mégevand et al., who found similar results between both arms, despite the higher donor site morbidity and flap failure with muscle flaps [28].

Another potential bias is the absence of standardization in the outcome reported between the studies. The definitions changed between authors and were not accounted for in the pooled analysis, leading to a potential over/underestimation of rates. The use of standardized scales such as the Clavien–Dindo classification for complications can ease the comparison between studies and avoid potential bias [29].

Overall, surgical outcomes appear to be favourable for FF and PF. Current knowledge tends to favour FFs, which are now often considered as the gold standard [4,8,30]. Despite the non-statistically significant differences, many outcomes assessed in this study tend to favour FF. FF offers the advantage of being able to cover large wounds, sometimes even with the use of chimeric flaps [31]. They offer different tissues from pliable to bulkier flaps. However, some orthoplastic surgeons prefer following the “reconstructive ladder” concept where PF can maintain a valuable option [32]. PF maintains a role when the defect is small because local flaps are rapid and reliable. Sometimes, even in larger wounds, PF can be favoured due to patient comorbidities or vascular damage (no recipient vessel).

While surgical outcomes have been widely discussed in this meta-analysis, their long term and functional outcomes must be investigated. Ducic et al. compared ambulation between PF and FF but did not find significant differences [14]. Other studies found similar functional outcomes between FF and PF in specific populations with variable overall results [33,34,35,36].

## 5. Conclusions

Despite its limitations, this meta-analysis identified that in cases where both options are available, FF and PF present similar surgical outcomes and are both valuable surgical strategies. We noted a trend in favour of FFs, which offers a valuable surgical strategy due to their vast choice of donor sites. Flap choice should be made depending on the defect aetiology, resources available, surgeon’s experience, and the patients’ comorbidities and expectations.

## Figures and Tables

**Table 1:** Search strategy.

Database	Search Strategy	N° of Articles
Pubmed	(free flap[MeSH Terms/Title/Abstract]) AND (pedicled flap[MeSH Terms/Title/Abstract]) AND ((lower limb[MeSH Terms/Title/Abstract]) OR (lower extremity[MeSH Terms/Title/Abstract])) AND ((comparative study[MeSH Terms/Title/Abstract]) OR (comparison[Title/Abstract]) OR (versus[Title/Abstract]) OR (vs[Title/Abstract]))	214
Embase/Medline/Preprints	(‘free tissue graft’:kw,ti,ab OR ‘free flap reconstruction’:kw,ab,ti) AND (‘pedicled skin flap’:ab,kw,ti OR ‘flap’:ab,kw,ti) AND ‘lower limb’:ab,kw,ti AND ‘comparative study’:ab,kw,ti	1
Cochrane library	ID Search Hits #1 MeSH descriptor: [Surgical Flaps] explode all trees #2 MeSH descriptor: [Free Tissue Flaps] explode all trees #3 MeSH descriptor: [Lower Extremity] explode all trees #4 (“pedicle flap”):ti,ab,kw OR (pedicled flap):ti,ab,kw (Word variations have been searched) #5 (free flap):ti,ab,kw (Word variations have been searched) #6 (“lower-limb”):ti,ab,kw OR (“lower extremity”):ti,ab,kw (Word variations have been searched) #7 #2 OR #5 #8 #1 OR #4 #9 #3 OR #6 #10 #7 AND #8 AND #9	13
Web of science	(((ALL = (free flap OR free tissue transfer)) AND ALL = (pedicled flap)) AND ALL = (lower limb OR lower extremity)) AND ALL = (comparative study OR comparison OR versus OR vs)	24

**Table 2:** Inclusion/exclusion criteria.

PICOS	Inclusion	Exclusion
Population	Adults and children who undergo lower limb reconstruction.	Cadaveric, animal studies. Upper extremity
Intervention	Free flap	
Comparator	Pedicled flaps (local, perforator, muscular, fascio-cutaneous)	
Outcomes	Main outcome flap necrosis, complications, patient satisfaction.	Studies that do not report main outcome
Study design	Comparative studies.	Reviews, meta-analysis, case reports, case series. Unpublished studies.

**Table 3:** Characteristics of the included studies.

Author	Year	Study Period	N° Patients	Mean Age FF (SD or Range)	Mean Age PF (SD or Range)	N° FF	N° PF	Type of Flaps (FF/PF)	Mean Follow-Up (SD or Range)	Mean Defect Surface FF cm^2^	Mean Defect Surface PF cm^2^
Abdelrahman [12]	2016	2012–2015	30	27.9 (9.7)	33.5 (10.6)	15	15	Muscle (FF)/FC flaps (PF)	***	35	28
Bhullar [21]	2020	2 years	40	36.7 (14.9)	48 (21.3)	17	23	Muscle flaps, FC flaps (FF)/Muscle flaps, FC flaps (PF)	33.8 m (20.6–49.5)	***	***
Cajozzo [13]	2017	2010–2015	37	61 (41–77)	74 (64–82)	20	17	Muscle flaps, FC flaps (FF)/FC flaps (PF)	***	***	***
Ducic [14]	2011	1990–2000	80	51 (32–75)	56 (44–77)	52	34	Muscle flaps, FC flaps (FF)/Muscle flaps (PF)	97.2 m (37.2)	***	***
Györi [15]	2022	***	34	54.9 (11.9)	62.4 (14.8)	13	21	Muscle flaps (FF)/Muscle flaps, FC flaps (PF)	Min 12 m	***	***
Innocenti [16]	2019	2009–2015	179	49 (5–89)	53 (11–92)	100	79	FC flaps (FF), FC flaps (PF)	12 m (***)	136	68
Koh [17]	2018	2011–2015	***	50.6 (17.4)	54.3 (19.2)	433	52	FC flaps (FF)/FC flaps (PF)	Min 12 m	***	***
Li [18]	2016	2007–2014	144	§	§	57	87	FC flaps (FF), FC flaps, Muscle flaps (PF)	***	***	***
Yuan [19]	2021	2010–2018	130	45.47 (***)	44.72 (***)	47	83	***	Min 6 m	81	49
Zook [20]	1986	1976–1982	58	***	***	41	23	Muscle flaps, FC flaps (FF)/Muscle flaps, FC flaps (PF)	***	***	***

FF = free flap/PF = pedicled flap/FC = fascio-cutaneous; § Median age of the whole population provided 37.9 (3–74); *** Data not reported in the study.

**Table 4:** Leave-one-out sensitivity test.

Pooled Risk Ratio				
Outcome	Removed Study	Estimate (95%CI)	*p*	I^2^ (%)
Total flap necrosis/flap loss	Abdelrahman [12]	1.37 (0.76–2.46)	0.30	0
	Bhullar [21]	1.24 (0.69–2.25)	0.47	0
	Cajozzo [13]	1.35 (0.76–2.39)	0.31	0
	Ducic [14]	1.28 (0.72–2.30)	0.40	0
	Györi [15]	1.29 (0.72–2.31)	0.39	0
	Innocenti [16]	1.30 (0.69–2.45)	0.41	0
	Koh [17]	1.38 (0.68–2.79)	0.37	0
	Li [18]	1.62 (0.87–3.00)	0.13	0
	Yuan [19]	1.43 (0.78–2.63)	0.25	0
	Zook [20]	1.29 (0.72–2.31)	0.39	0
Partial flap necrosis	Abdelrahman [12]	0.45 (0.20–1.02)	0.06	29
	Cajozzo [13]	0.37 (0.16–0.83)	0.02	14
	Ducic [14]	0.42 (0.19–0.94)	0.04	29
	Györi [15]	0.41 (0.19–0.86)	0.02	21
	Innocenti [16]	0.59 (0.31–1.12)	0.11	0
	Li [18]	0.52 (0.26–1.06)	0.07	11
	Yuan [19]	0.38 (0.15–0.95)	0.04	26
	Zook [20]	0.43 (0.19–0.96)	0.04	29
Overall complication rate	Bhullar [21]	0.71 (0.52–0.97)	0.03	0
	Cajozzo [13]	0.83 (0.63–1.11)	0.21	10
	Györi [15]	0.82 (0.61–1.09)	0.16	12
	Innocenti [16]	0.83 (0.60–1.14)	0.25	13
	Yuan [19]	0.91 (0.69–1.20)	0.50	0
	Zook [20]	0.84 (0.63–1.14)	0.26	9
Revision surgery rate	Bhullar [21]	1.46 (0.43–5.00)	0.55	85
	Cajozzo [13]	1.53 (0.52–4.53)	0.44	86
	Györi [15]	1.32 (0.41–4.21)	0.64	86
	Innocenti [16]	1.81 (0.68–4.78)	0.23	80
	Yuan [19]	0.94 (0.58–1.52)	0.80	8
High aesthetic satisfaction rate	Cajozzo [13]	1.37 (0.4–4.63)	0.61	96
	Ducic [14]	2.33 (1.56–3.46)	<0.05	0
	Yuan [19]	1.70 (0.16–17.85)	0.66	91
Post-operative wound infection rate	Bhullar [21]	0.72 (0.40–1.28)	0.26	0
	Cajozzo [13]	0.87 (0.56–1.36)	0.55	0
	Li [18]	0.91 (0.52–1.59)	0.74	0
	Yuan [19]	0.88 (0.54–1.44)	0.62	0
	Zook [20]	0.86 (0.55–1.35)	0.50	0

## Data Availability

Not applicable.
